# Supplementary material for: A Receptor Integrin β1 Promotes Infection of Avian Metapneumovirus Subgroup C by Recognizing a Viral Fusion Protein RSD Motif
Source: Int J Mol Sci. 2024 Jan 9;25(2):829. doi: 10.3390/ijms25020829 (PMC10815723; doi:10.3390/ijms25020829)
Supplement: Supplementary file 1 [file ijms-25-00829-s001.zip › Supplementary_material.pdf]

Supplementary Figure Captions

Figure S1. Relationship between different metapneumovirus was deduced using the F genes. The ML tree was reconstructed using IQtrees. The different metapneumovirus are expressed by different colors. The conserved integrin-binding motifs located in the F protein were all labeled behind the phylogenetic tree.

Figure S2. (A) F protein structure of aMPV/C predicted using AlphaFold2. The F protein includes three regions, F0, F1, and F2, where F0 is the splicing region. (B) Location of the RSD motif in the F protein.

Figure S3. F protein structures of hMPV and aMPV/C. A comparison of the structures of the two viruses predicted by Alphafold2.

Figure S4. aMPV/C F protein can bind with hITGB1. POCASA 1.1 was used to predict the binding sites, and AutoDock Vina1.1.2 was used for docking.
