# Supplementary figures and images for: A Receptor Integrin β1 Promotes Infection of Avian Metapneumovirus Subgroup C by Recognizing a Viral Fusion Protein RSD Motif
Source: Int J Mol Sci. 2024 Jan 9;25(2):829. doi: 10.3390/ijms25020829 (PMC10815723; doi:10.3390/ijms25020829)

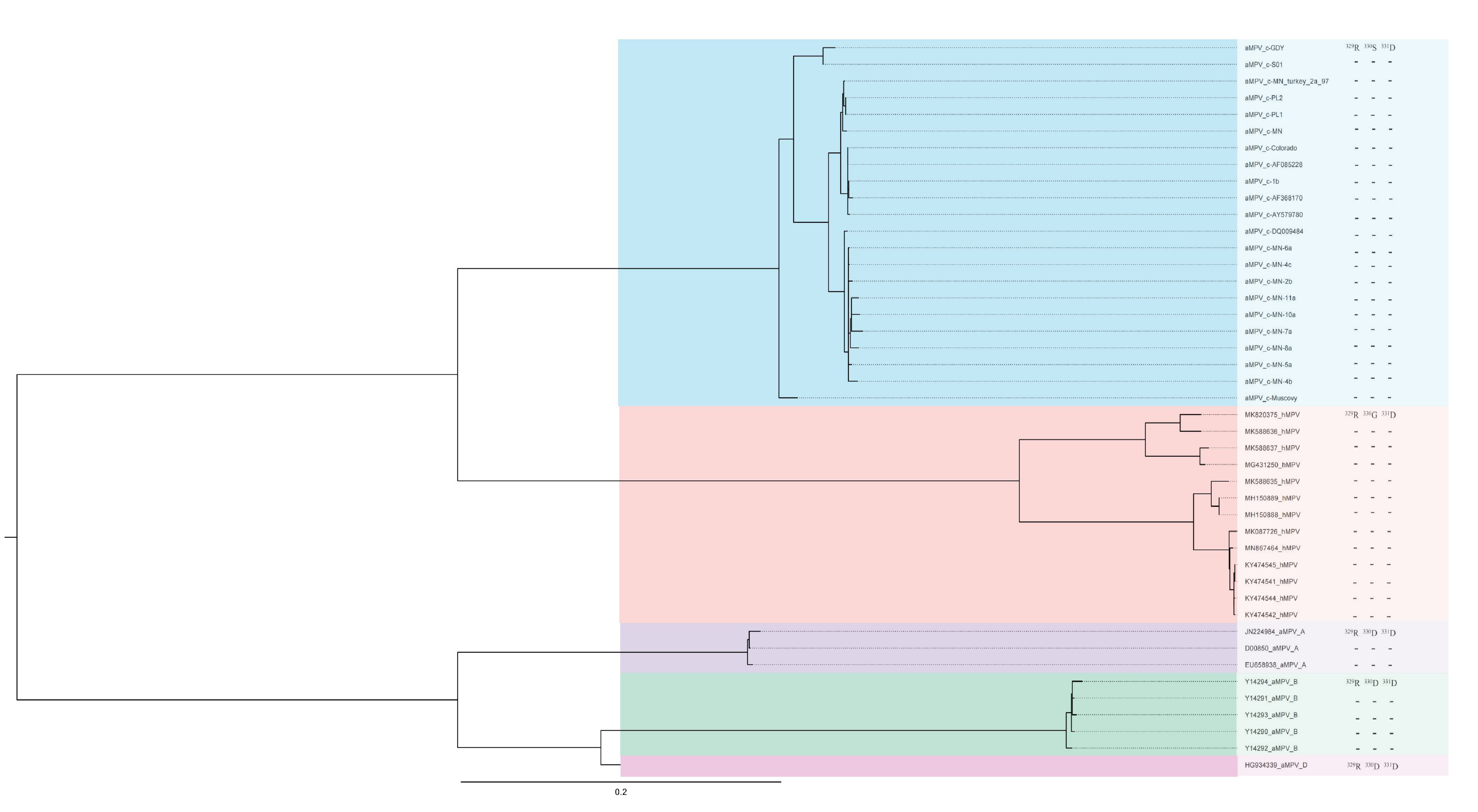

Supplement: Supplementary file 1 [file ijms-25-00829-s001.zip › Fig.S1.tif]

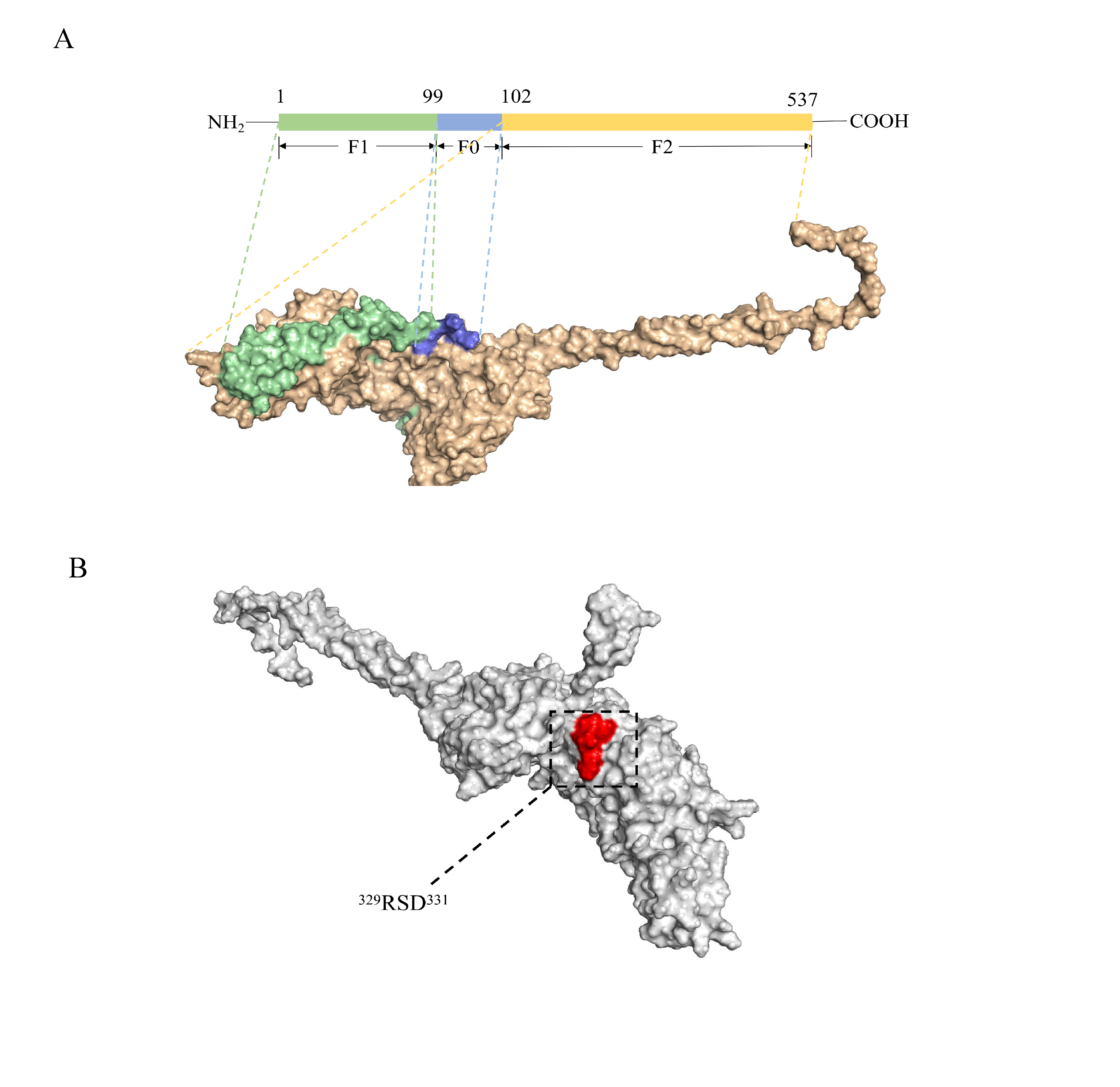

Supplement: Supplementary file 1 [file ijms-25-00829-s001.zip › Fig.S2.tif]

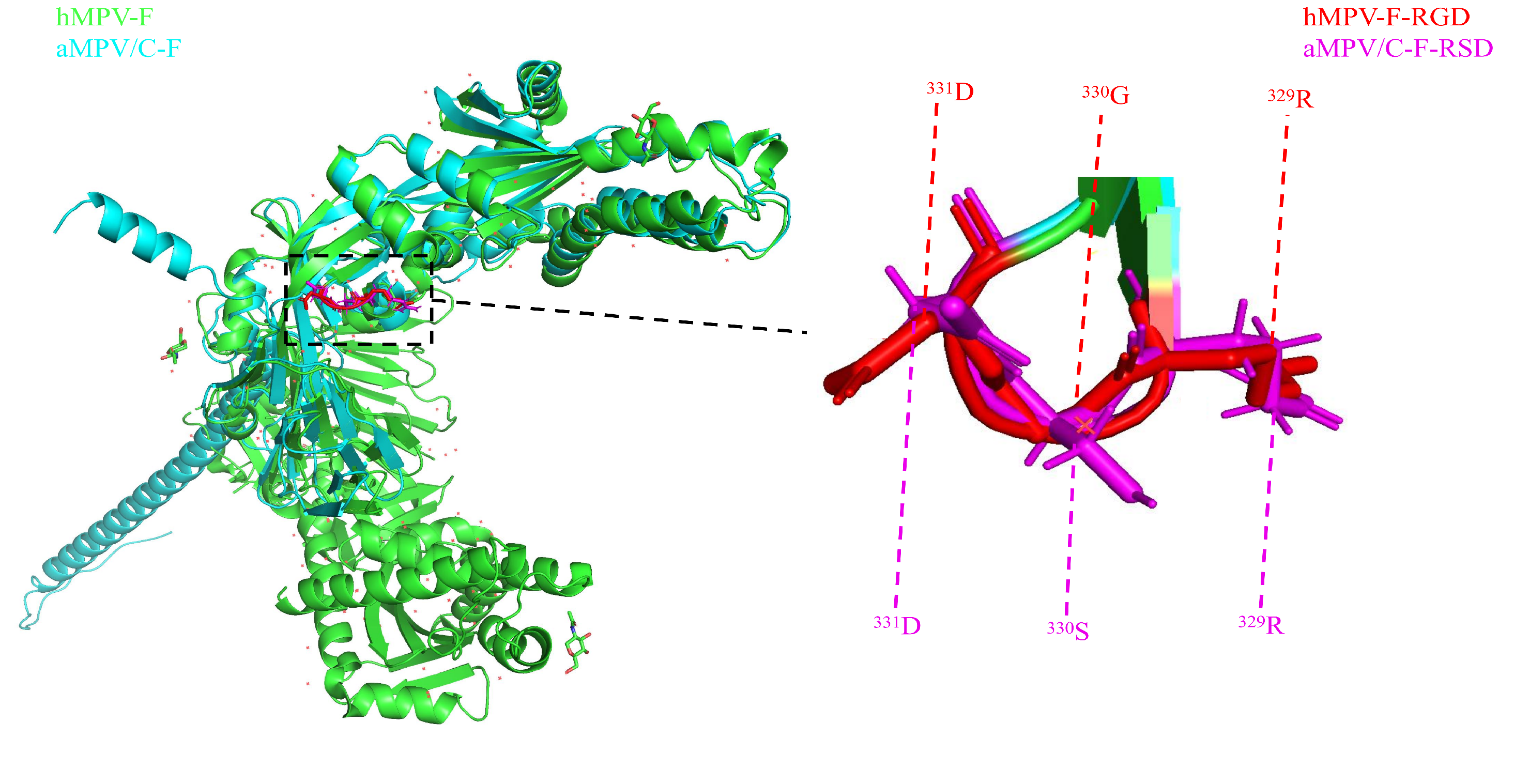

Supplement: Supplementary file 1 [file ijms-25-00829-s001.zip › Fig.S3.tif]

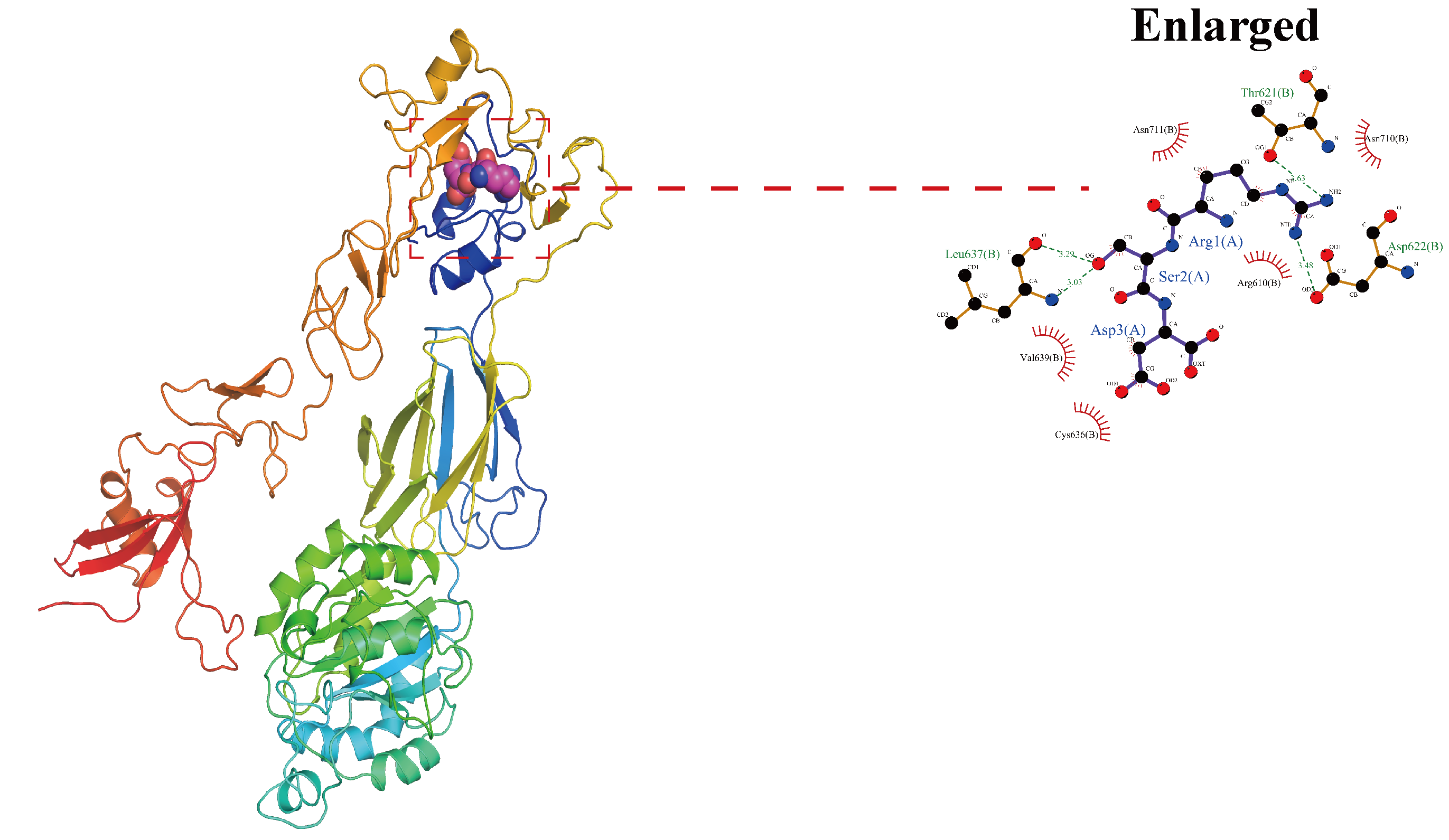

Supplement: Supplementary file 1 [file ijms-25-00829-s001.zip › Fig.S4.tif]
